# Cardiac Explant-Derived Cells Are Regulated by Notch-Modulated Mesenchymal Transition

**DOI:** 10.1371/journal.pone.0037800

**Published:** 2012-05-25

**Authors:** Liudmila Zakharova, Hikmet Nural-Guvener, Mohamed A. Gaballa

**Affiliations:** 1 Center for Cardiovascular Research, Banner Sun Health Research Institute, Sun City, Arizona, United States of America; 2 Internal Medicine, University of Arizona, Tucson, Arizona, United States of America; Centro Cardiologico Monzino, Italy

## Abstract

**Background:**

Progenitor cell therapy is emerging as a novel treatment for heart failure. However the molecular mechanisms regulating the generation of cardiac progenitor cells is not fully understood. We hypothesized that cardiac progenitor cells are generated from cardiac explant via a process similar to epithelial to mesenchymal transition (EMT).

**Methods/Findings:**

Explant-derived cells were generated from partially digested atrial tissue. After 21 days in culture, c-Kit+ cells were isolated from cell outgrowth. The majority of explant-originated c-Kit+ cells expressed the epicardial marker Wt1. Cardiac cell outgrowth exhibits a temporal up-regulation of EMT-markers. Notch stimulation augmented, while Notch inhibition suppressed, mesenchymal transition in both c-Kit+ and c-Kit- cells. In c-Kit+ cells, Notch stimulation reduced, while Notch inhibition up-regulated pluripotency marker expressions such as Nanog and Sox2. Notch induction was associated with degradation of β-catenin in c-Kit- cells. In contrast, Notch inhibition resulted in β-catenin accumulation, acquisition of epitheloid morphology, and up-regulation of Wnt target genes in c-Kit- cells.

**Conclusion:**

Our study suggests that Notch-mediated reversible EMT process is a mechanism that regulates explant-derived c-Kit+ and c-Kit- cells.

## Introduction

Heart failure after myocardial infarction (MI) is a major public health issue worldwide. To date, heart transplantation remains the gold standard for treatment of end-stage heart failure, a procedure that is limited by donor availability. Cell-based therapy is emerging as a novel alternative for the treatment of heart failure. Recently, we and others have demonstrated that cardiac-explant-derived cells can be generated directly from cardiac biopsies [1]–[4]. Transplantation of these cells improved cardiac function after MI [2]. Understanding the molecular mechanisms that controls the process of cell outgrowth from cardiac explants will assist the development of more efficient cell-based therapy. For a cell to become mobile it must undergo a mesenchymal transition [5]. A well documented form of mesenchymal transition is the epithelial/endothelial to mesenchymal transition (EMT). EMT is a key step during embryonic morphogenesis, and is reactivated as a response to tissue injury and tumor formation in adults [6], [7]. Repression of E-cadherin/VE-cadherin by transcriptional regulators such as Snail and Slug, (both are members of Snail repressors family) are the critical step in driving EMT. During development, the transition of epithelial to mesenchymal phenotype is reversible as several rounds of EMT and mesenchymal to epithelial transition (MET) are required for the final differentiation of cells. For instance, the heart forms through three successive cycles of EMT and MET [5]. During development, epicardial EMT was shown to generate cardiovascular progenitor cells that differentiate into cardiomyocytes [8]. In adults, EMT occurs as a physiological response to injury. For example, during wound healing, keratinocytes recapitulate part of the EMT process resulting in acquisition of an intermediate phenotype, which allows ketarinocytes to migrate [9]. More relevant to the current study, myocardial injury induced re-activation of epicardial cells via EMT; these cells migrated to the site of injury and contributed to cardiac regeneration [10], [11]. Post-injury epicardial EMT was associated with the re-expression of embryonic markers such as Tbx18 and Wt1 [10], [12]. At the cellular level, pathological and physiological EMTs were similar, in that they were governed by similar signaling pathways, regulators, and effector molecules. These pathways include TGF-β, Wnt/β-catenin, Notch, Hedgehog, and others [13], [14]. Among them, the Notch pathway appeared to promote cardiac gene expression and myocyte differentiation [15], [16]. A similar procardiogenic action of Notch had been reported in mesenchymal cells, which was possibly a reiteration of the EMT that occurred during embryonic cardiac development [17].

Here we determined that explant-derived cells undergo EMT-like changes in culture. Our data demonstrated that mesenchymal phenotype of explant derived cells is reversible, and is regulated by Notch signaling. We also showed that pluripotent gene expressions in c-Kit+ cells are regulated by Notch. Overall our findings suggest that Notch signaling molecules could be used to modulate cardiac outgrowth phenotype in vitro. These new insights into the molecular mechanisms of the cardiac progenitor cells regulation in vitro will help define the development of a more efficient cell-based therapy for heart failure.

## Materials and Methods

### Generation of Explant-derived Cells

Animal studies were performed in a facility accredited by American Association for Accreditation of Laboratory Animal Care. Animal studies were performed in accordance with federal laws and regulations, international accreditation standards, and institutional policies including approval by the Animal Care and Use Committee of Banner Sun Health Research Institute (IACUC protocol #10-03). Atrial tissue was obtained from 3 month old male Harlan Sprague Dawley rats. Cardiac explant outgrowth was generated as previously described [1], [2]. Briefly, tissue was cut into 1–2 mm^3^ pieces and digested with 0.2% trypsin (Invitrogen, Carlsbad, CA) and 0.1% collagenase IV (Invitrogen) for 10 minutes. The remaining tissue fragments were cultured as “explants” in explants medium (EM) which was composed of IMDM supplemented with 10% fetal bovine serum (FBS), 100 U/ml penicillin G, 100 µg/ml streptomycin, 2 mmol/L L-glutamine, and 0.1 mmol/L 2-mercaptoethanol. For the time course experiments, digested tissue was placed one piece per well into poly-L-lysine (PLL)-coated cell culture plates to facilitate adhesion. After a few days in culture, a layer of cardiac stromal cells grew out of the adherent explants, over which small phase-bright cells were formed. After 21 days in culture, Cells were collected by pooling two washes of Ca^2+^-Mg^2+^-free PBS supplemented with 0.53 mmol/L EDTA and one wash of 0.25% Trypsin-0.53 mmol/L EDTA in PBS for 5 min. Combined washes were centrifuged at 200×g for 5 min and the cell pellet was collected. C-Kit+ population was separated from the cell outgrowth using magnetic beads (MACS, Miltenyi Biotec) according to manufacturer protocol and was analyzed by flow cytometry to confirm the purity.

### Transfection of Notch intracellular Domain (NICD)

Freshly isolated c-Kit- and c-Kit+ cells were plated separately at 1×10^5^ cells per well in EM media in 12 well-plates. The next day cells were transfected with an adenovirus vector coding Notch intracellular domain (NICD) and enhanced green fluorescent protein (eGFP) (generous gift of Dr. M. Sussman, University of California; originally created by Dr. Tezuka [18]). Control cells were transfected with adenovirus carrying eGFP only. Transfection efficiency of >90% was confirmed by GFP expression. Cells were collected at day 7 after transfection.

### Cell Treatment with γ-secretase Inhibitor

Freshly isolated c-Kit- and c-Kit+ cells were plated at 1×10^5^ cells per well in EM media. γ-Secretase inhibitor XXI (GSI) (Calbiochem) was dissolved in DMSO and was added to cell culture media at the final concentration of 10 nM. Cells were treated for 7 days. Control cells were treated with DMSO (0.005% final concentration).

### Immunostaining and Cell Quantification

For the time course experiments, explants were cultured for 4, 8 and 21 days. Explant tissue was removed and cells were fixed/permeabilized in a 1∶1 mixture of acetone/100% ethanol. For c-Kit surface labeling cells were fixed with 4% paraformaldehyde. Cells were blocked with 3% BSA in PBS and stained with primary antibody (Table S1). Corresponding secondary antibodies were conjugated with Alexa-488 or Alexa 568 (Molecular Probes). Nuclei were stained with DAPI, 4′ 6-diamidino-2-phenylindole (Invitrogen).

For cell quantification, the number of cells was counted in ten random microscopic fields for each antigen. The percentage of antigen-positive cells was calculated as a number of positively-stained cells normalized to the total number of cells. For the 8 and 21 day time points, at least 2000 cells total were counted in each case. For the 4-day time point, 200–600 cells were counted in each case due to the low number of explant-derived cells at this early time point.

### Immunostaining of Heart Sections

Heart tissue was embedded in tissue frozen media (Triangle Biomedical Science) snap-frozen in liquid nitrogen and sectioned coronally using Leica CM1900 cryostat (Leica Microsystems, Bannockburn, IL). Coronal tissue sections (5–7 µm thickness) were mounted on positively charged glass slides, and fixed/permeabilized in a 1∶1 mixture of acetone/100% ethanol. For immunofluorescent staining, fixed tissue sections were blocked with 3% BSA in PBS and incubated with primary antibodies (Table S1). Specific staining was visualized using corresponding secondary antibodies conjugated with Alexa 488 or Alexa 568 (Molecular Probes).

### RNA Isolation and Quantitative Real-time RT-PCR

Total RNA was extracted from total outgrowth, c-Kit+ or c-Kit- cells using RNAspin mini isolation kit (GE Healthcare). RNA integrity and concentration were assessed on the Agilent 2100 Bioanalyzer with RNA 6000 Nano LabChip kit (Agilent Technologies Inc). Total RNA (100 ng) was reverse transcribed with QuantiTect Reverse Transcription kit (Qiagen). Real time RT-PCR was conducted using the Rower SYBR Green Master Mix (Applied Biosystems) on a StepOnePlus Real-time PCR System (Applied Biosystems). Specific primers were synthesized by Invitrogen (sequences are available upon request). β-Actin was used as a reference gene. Data analysis was performed on StepOne software version 2.1 (Applied Biosystems) using the comparative Ct ( ΔΔCt) quantitation method.

### Western Blotting

Cells were lysed in RIPA buffer (Thermo Scientific) containing Halt Phosphatase and Proteinase inhibitor cocktail (Thermo Scientific) according to the manufacturer protocol. Protein concentration was determined using BCA Protein Assay kit (Thermo Scientific). Equal amount of protein (50 µg) was loaded in each well of 10% Tris-Glycine gel (Bio-Rad Laboratories) and subjected to electrophoresis. Proteins were transferred to PVDF membrane (Invitrogen), and then blocked with 5% non-fat dry milk in Tris buffered saline (TBS) followed by overnight incubation with primary antibodies at 4°C. Membranes were washed in TBS containing 0.05% Tween 20. Corresponding horse radish peroxidase (HPR)-conjugated anti rabbit or anti mouse IgGs (Invitrogen) were used as secondary antibodies. Blots were probed with an anti β-actin antibody as a loading control. Immunoreactive proteins were detected by chemiluminescence (Thermo Scientific). Band intensity was determined using FluorChem 8900 software (Alpha Innotech Corp).

### Flow Cytometry

Cells were fixed in 70% ethanol and labeled with the following antibodies: c-Kit, E-cadherin (Santa-Cruz Biotechnology), MHC, SMA, (Abcam) and cleaved caspase 3 (Cell signaling Tech). Cells were treated with secondary antibodies corresponding to either anti-rabbit or anti-mouse IgG conjugated with Alexa-488, phycoerythrin (PE) or PE-Cy5.5 (Invitrogen). For a negative control, cells were labeled with isotype IgG instead of primary antibody. Cell events were detected using FACS Calibur flow cytometer equipped with argon laser (BD Biosciences). Data was analyzed using CellQuest software (BD Biosciences).

### Proliferation Assay

Cells were labeled with 10 µM BrdU for 40 min. Cells were harvested and fixed with 70% ethanol, following with the treatment of 30 µg DNAse I for 1 h to denature DNA, and then incubated with anti-BrdU antibody conjugated with Fluorescein isothiocyanate (FITC). Cell events were detected using FACS Calibur flow cytometer equipped with argon laser (BD Biosciences). Data was analyzed using CellQuest software (BD Biosciences).

### Imaging

Images were captured at room temperature using Olympus IX-51 epifluorescence microscope equipped with a DP72 device camera. The following excitation/emission maximum filters: 490 nm/520 nm, 570 nm/595 nm and 355 nm/465 nm, were used for image acquisition. Images were processed using DP2-BSW software (Olympus Corp). Acquisition settings were held constant within each experiment.

### Statistics

All data was represented as mean ± S.E. Significance (p<0.05) was determined using Student’s t-test for unpaired samples or directional Wilcoxon test. Statistical analysis was conducted using SigmaStat 3.5 software.

## Results

### Mesenchymal Transition of Cardiac Cell Outgrowth in Culture

**Figure 1:**
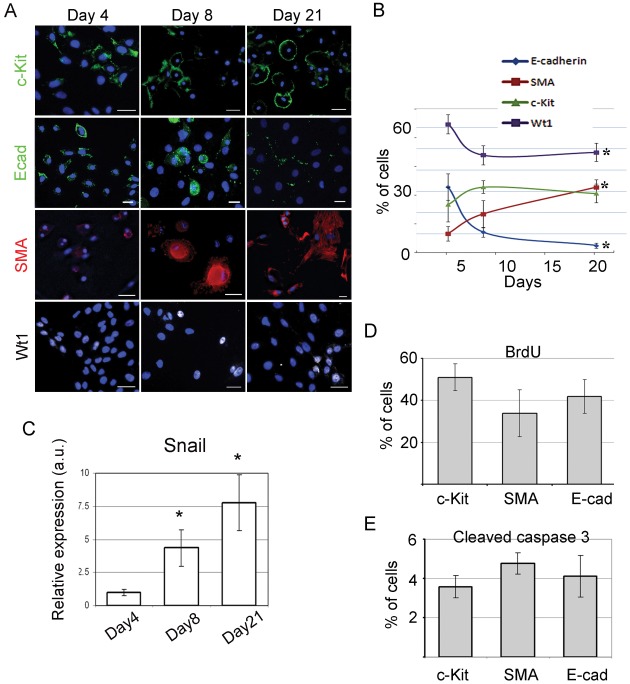
Expression of cell-specific markers in cardiac cell outgrowths is time-dependent. (**A**) Cell outgrowths were cultured for various times and analyzed by immunofluorescence for the expression of cell lineage markers: stem cell marker, c-Kit; cardiac structural proteins, α-myosin heavy chain (MHC); mesenchymal cell marker, α-smooth muscle actin (SMA), epithelial cell marker, E-cadherin (E-cad) and epicardial progenitor marker Willms tumor 1 (Wt1). Nuclei were counterstained with DAPI (blue). (**B**) Percentage of cells expressing cell lineage markers. * p<0.05, compared to day 4. (**C**) Temporal changes of Snail gene expression were measured by qRT-PCR; *, p<0.05 compared to day 4. (**D**) Proliferation of outgrowth sub-populations. Proliferating (BrdU-positive) marker-specific outgrowth sub-populations were detected by flow cytometry as double positive events. The percentage of marker-specific proliferating cells was calculated as number of BrdU+/marker+ cells normalized to the total number of marker+ cells. (**E**) Apoptosis of cell sub-populations. Apoptotic (cleaved caspase 3-positive) marker-specific outgrowth sub-populations were detected by flow cytometry as double positive events. The percentage of marker-specific apoptotic cells was calculated as a number of caspase-3+/marker+ cells normalized to the total number of marker+ cells.

Cardiac explant outgrowth was generated from partially digested atrial tissue as previously reported by our laboratory and others [1]–[4]. We monitored temporal changes in EMT markers in these cells at 4, 8, and 21 days in culture using immunocytochemistry (Figure 1 A, B). We found that the percentage of cells expressing epithelial markers E- cadherin (E-cad) and Wilms tumor 1 (Wt1) [11], [19] decreased in culture (32±6.2% vs. 4.5±1.2% for E-cad, and 61.5±5.3% vs. 49±3.% for Wt1 at day 4 vs. day 21, respectively). The percentage of cells expressing mesenchymal cell marker α-smooth muscle actin (SMA) increased (from 10.1±3.4% to 31.9±3.5% at day 4 vs. day 21 respectively), while, the percent of c-Kit+ progenitor cells remained relatively unchanged during the indicated times (23.8±8.1% vs. 29±4.4% day 4 vs. day 21). In addition, gene expression of Snail, a well-described EMT marker, was increased with time in culture (Figure 1C). Taken together, our data suggest that cardiac cell outgrowth undergo an EMT-like process in culture. To determine whether this temporal increase in mesenchymal marker may be due to selective high proliferation and/or diminished apoptosis of fibroblasts, we measured both cell proliferation and apoptosis. After 21 days in culture, BrdU incorporation rate was similar for c-Kit+, E-cad+ and SMA+ cells (51.0±6.4%, 41.8±8.4%, and 34.0±11.1% respectively) (Figure 1D, Figure S1) suggesting that fibroblast proliferation rate did not exceed that of other cell sub-population in culture. We also examined the level of apoptosis on cell outgrowth by double-labeling with antibodies to cell–specific markers and to activated (cleaved) caspase-3. All studied cell sub-populations displayed similar levels of cleaved caspase 3 (3.5±0.5%, 3.6±1.6% and 4.5±0.6%, for c-Kit+, E-cad+, and SMA+ cells respectively) (Figure 1E, Figure S1). Collectively, these results suggested that the temporal increase in mesenchymal markers is unlikely due to selective proliferation and/or diminished apoptosis of fibroblasts but rather due to EMT.

### Characterization of Explant-originated c-Kit+ Cells

**Figure 2:**
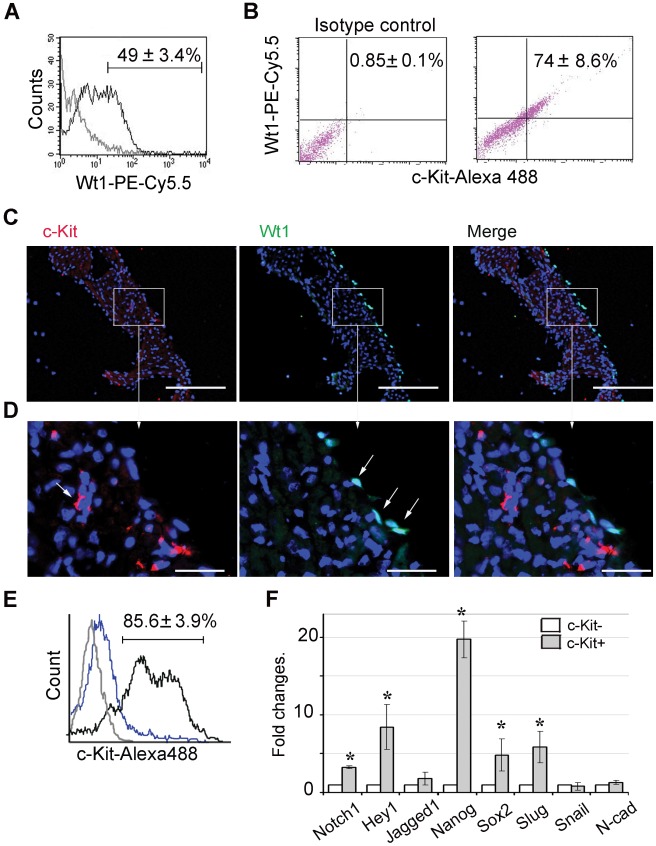
Characterization of c-Kit+ cells isolated from total outgrowths. (**A**) Flow cytometry analysis of outgrowths cultured for 21 days shows percent of Wt1+ cells. (**B**) FACS analysis of double c-Kit and Wt1 labeling. Percentage of c-Kit+/Wt1+ cells indicated in upper right quadrant of histogram was calculated as a ratio of c-Kit+/Wt1+ (UR) to the total number of c-Kit+ cells (UR + LR). (**C–D**) Heart coronal sections are labeled with anti c-Kit (red) or anti Wt1 (green) antibodies. Nuclei were stained with DAPI (blue). Right column represents merged images of consecutive sections labeled with c-Kit and Wt1. (**C**) Representative images of left atrium are shown. Scale bar, 100 µm. (**D**) Higher magnification of the area selected in the box. Scale bar, 20 µm. (**E**) Purity of c-Kit+ and c-Kit- cell populations were confirmed by FACS analysis. C-Kit+ (black) and c-Kit- (blue) cells were labeled with anti-c-Kit antibody followed by secondary antibody conjugated with Alexa 488. For negative control, isotype IgG was used instead of primary antibody (grey). Representative histogram is shown. (**F**) qRT-PCR analysis of c-Kit+ and c-Kit- cell populations. Expression levels were normalized to the level of β-actin, fold changes were calculated as a ratio of expression in c-Kit- group to expression in c-Kit+ group, * p<0.05.

Using flow cytometry, we found 74+8.6% of c-Kit+ cells, while only 49+3.4% of total cardiac outgrowth expressed Wt1, suggestive of epicardial origin (Figure 2A,B) [11], [19]. Next we questioned whether or not Wt1+/c-Kit+ cells are present in atrial tissue in vivo. To answer that, whole heart coronal sections were analyzed by immunohistochemistry. We found that Wt1+ cells were located in a thin layer of epicardial cells of both right and left atria of normal hearts (Figure 2C–D), while c-Kit+ cells were found primarily within the sub-epicardium and myocardium albeit at low frequency (Figure 2C–D). No overlap was observed between c-Kit+ and Wt1+ cells in the atrium (Figure 2C–D). This result indicates that epicardial Wt1+ cells within the atrium do not express c-Kit marker in vivo, but more likely they acquired c-Kit+ phenotype in culture.

Next, to determine if c-Kit+ and c-Kit- cells expressed EMT and pluripotency markers differently in culture, we sorted cell outgrowth based on c-Kit expression at 21 days in culture. Purity of c-Kit+ and c-Kit- cells was confirmed by FACS and immunocytochemistry (Figure 2E and Figure S2, respectively). Gene expression profile of c-Kit+ and c-Kit- cell populations were analyzed by qRT-PCR. We found c-Kit+ cells expressed higher levels of the EMT marker Slug (also known as Snail 2) and pluripotency markers Nanog and Sox2 (Figure 2F), while no differences were found in other EMT markers, Snail and N-cadherin (N-cad) compared to c-Kit- cells (Figure 2F). These data indicate that both c-Kit- and c-Kit+ cells underwent EMT-like process in culture. Furthermore, c-Kit+ cells expressed significantly higher gene expression levels of Notch1 and Hey1, a direct down-stream target of Notch, compared to c-Kit- cells (Figure 2F) [20].

**Figure 3:**
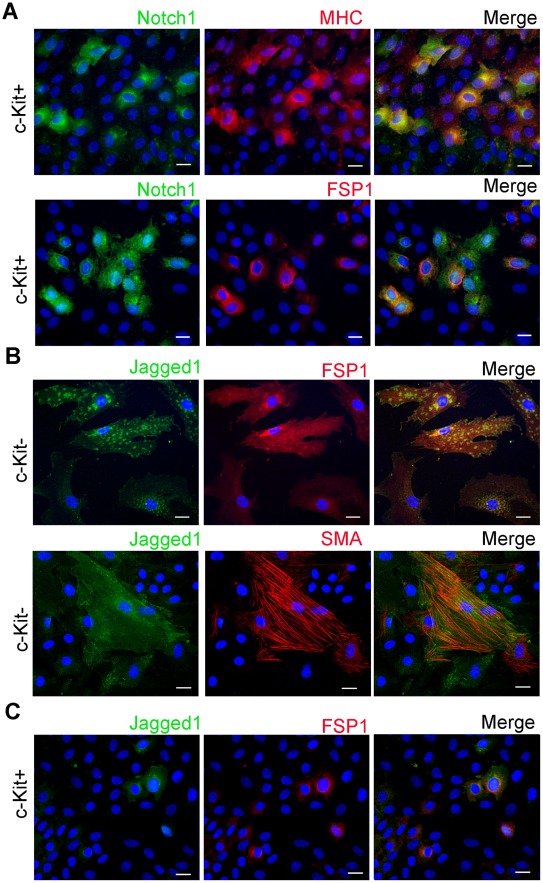
Expression of Notch1 receptor and Jagged1 in c-Kit+ and c-Kit- cells. (**A**) c-Kit+ cells displayed positive staining for Notch1 (green) co-localized with MHC (red, top row) or FSP1 (red, bottom row). (**B**) C-Kit- cells displayed positive staining for Jagged (green) co-localized with FSP1 (red, top row) or SMA (red, bottom row) as indicated. (**C**) Low number of c-Kit+ cells also express Jagged1 (green) co-localized with FSP1 (red). (**A–C**) Scale bar, 20 µm.

Next, to determine whether or not Notch-active c-Kit+ cells may interact with c-Kit- cells in culture, we labeled c-Kit+ and c-Kit- cells with either Notch1 or Jagged1. We found that Notch1 was mainly expressed in c-Kit+ population, while Jagged1 was mainly observed in c-Kit- cells (Figure 3 A–C). Notch1+/c-Kit+ cells were also positive for either myosin heavy chain (MHC) or fibroblast specific factor 1 (FSP1), while in c-Kit- cells, Jagged1 was co-localized with either FSP1 or SMA, (Figure 3 A–C). These data suggest that Notch1+/c-Kit+ cells in cardiac outgrowth may be stimulated by cell contacts with Jagged1+/c-Kit-, which is consistent with previous reports indicating that Notch requires a direct contact between a receptor-expressing and a ligand-expressing cell to elicit an inductive signal [21].

### Notch Suppression Delays Cardiac Explant Outgrowth

Notch signaling is known to regulate EMT of epicardial cells [22], therefore to determine whether or not EMT plays a role in c-Kit+ generation within cardiac cell outgrowth, we suppressed EMT using Notch inhibitor, γ-secretase inhibitor XXI (GSI) and examined the effects on cell outgrowth and c-Kit+ formation. We observed that GSI treatment resulted in significant delay in cell outgrowth compared to control. At 4 days in culture, 85% of control explants and 20% of GSI-treated explants (out of 20 examined) attached to the plate and started to generate cells (Figure S3). By 8 days, all of the control explants attached and produced large number of cells, while only 75% of GSI-treated explants attached and produced significantly less cells compared to controls. After 21 days in culture, cells generated in the presence of GSI exhibited epithelioid cobblestone-like morphology (Figure S3). GSI treatment tend to increase the number of c-Kit+ cells in cell outgrowth (from 22.8±7% to 40±13.6% control vs. GSI-treated) and Wt1+ cell number (from 39±6.3% to 54.1±7.4% control vs. GSI-treated; Figure S4). Taken together these data indicate that Notch suppression delays outgrowth, promotes epithelial morphology, and tend to increase the formation of c-Kit+ cells in culture.

### Notch Signaling Regulates Mesenchymal Transition of c-Kit+ and c-Kit-cells

**Figure 4:**
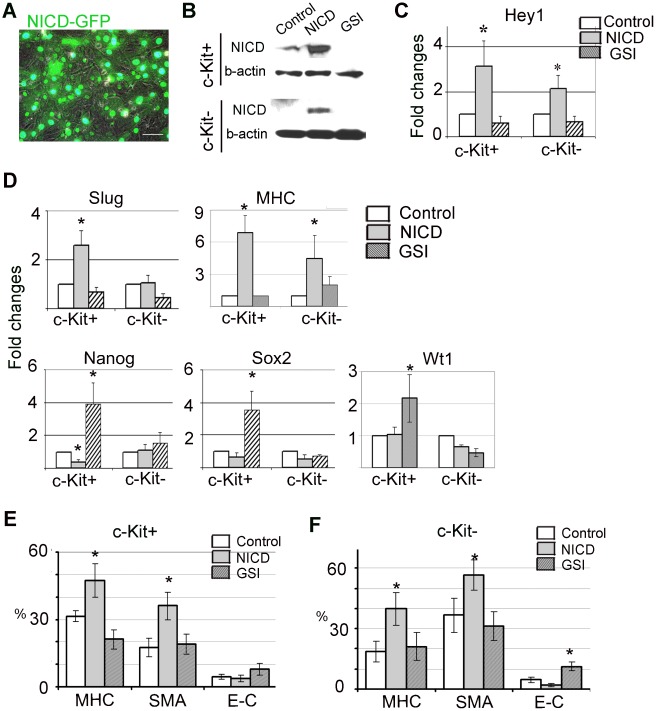
Notch signaling regulates mesenchymal transition of c-Kit+ and c-Kit- cells. Notch signaling was up-regulated by over-expressing NICD-GFP adenovirus or suppressed with γ-secretase inhibitor XXI (GSI). (**A**) NICD transfection efficiency was monitored by GFP expression. (**B**) Notch modulation was confirmed by a Western blot analysis with anti-NICD antibody and (**C**) by qRT-PCR measuring expression level of Hey1. (**D**) qRT-PCR expression analysis after Notch signaling modulation. Expression levels were normalized to the level of β-actin. Fold changes were calculated as a ratio of expression in experimental (NICD- or GSI-treated) group to the expression in control group, * p<0.05. (**E, F**) FACS analysis of c-Kit+ (**E**) and c-Kit- (**F**) cells after Notch signaling modulation. N = 5 per group, *, p<0.05 compared to control.

**Figure 5:**
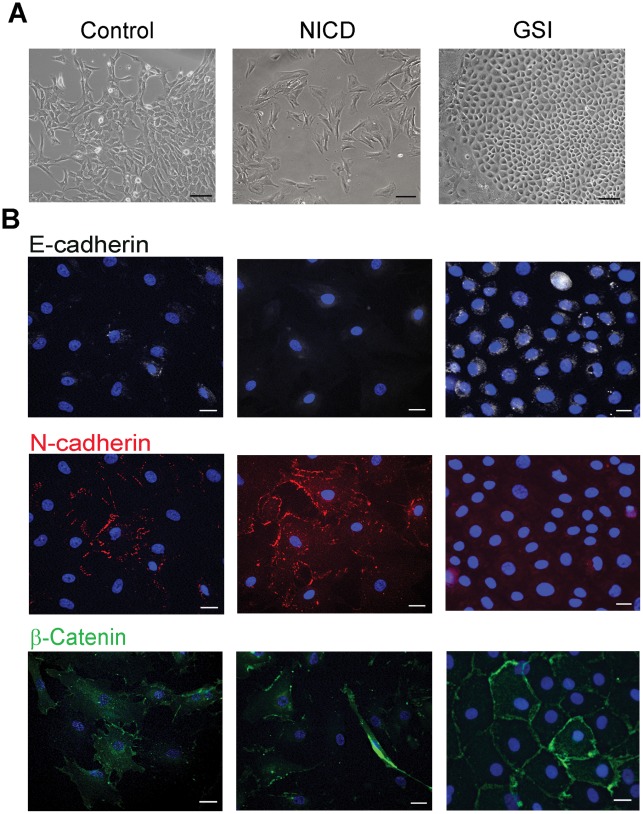
Suppression of Notch reversed mesenchymal phenotype of explant-derived cells. C-Kit+ and c-Kit- cells were cultured in presence of GSI. Representative images of c-Kit- cells are shown. (**A**) Transmitted light images demonstrate changes in cell morphology upon treatment with NICD or GSI. (**B**) Cells were labeled with antibodies to E-cadherin (white), N-cadherin (red) or β-catenin (green) as indicated. Nuclei were counterstained with DAPI (blue). Scale bars, 100 µm (A) or 20 µm (B).

To determine whether or not Notch signaling also regulate mesenchymal transition in c-Kit+ and c-Kit- cells, Notch was stimulated by adenoviral mediated gene transfer of Notch intracellular domain fused with GFP (NICD-GFP) and inhibited by GSI in both cell populations (Figure 4A). Modulation of Notch was validated by immunostaining, Western blot and qRT-PCR. Western blot analysis with activated Notch intracellular domain antibody (Val1744) showed that the amount of NICD is significantly increased in virus-transfected cells (Figure 4B). NICD was undetectable in control c-Kit- cells or in cells treated with GSI. Quantitative RT-PCR analysis confirmed that the expression level of down-stream Notch target gene Hey1 was significantly increased in NICD-transfected cells (Figure 4C). We found that in c-Kit+ cells, NICD over-expression increased Slug, while decreased Nanog gene levels. In contrast, suppression of Notch signaling significantly up-regulated expression of Nanog, Sox2 and Wt1 (Figure 4D). However, the cardiomyocyte marker MHC increased in both c-Kit+ and c-Kit- cells. (Figure 4D). FACS analysis showed that NICD over-expression increased SMA-positive cells (17±4.1% to 35±6.1% in c-Kit+ cells, and from 36±6.1% to 56.6±7.4% in c-Kit- cells, control vs. NICD-over-expressed respectively) and MHC-positive cells (31.0±2.4 to 47.3±7.4% in c-Kit+ cells and 18.7±5.1 to 39.8±8.2% in c-Kit- cells). The number of E-cadherin-positive cells was moderately decreased by Notch stimulation (4.5±1.25% to 3.8±1.4% in c-Kit+ cells, and 4.6±1.4% to 2.0±0.7% in c-Kit- cells) (Figure 4E, F; Figure S5, Tables S2 and S3). In contrast, Notch suppression increased E-cadherin-positive cells in both c-Kit+ and c-Kit- populations (4.5±1.25% to 7.9±2.6% in c-Kit+ cells and 4.6±1.4% to 11.2±2.2% in c-Kit- cells). GSI treatment decreased MHC-positive cells in c-Kit+ population (31±2.4% to 21.1±4.2%, control vs. GSI-treated) and had no effect on c-Kit- cells (18.7±5.1 vs. 21.2±7.0%). The number of SMA-positive cells remains relatively unchanged in GSI-treated cells compared to control (17.5±4%. vs.18.9±4.49% in c-Kit+ cells and 36±6.1% vs. 31.3±7.2% in c-Kit- cells ) (Figure 4E, F; Figure S5, Tables S2 and S3). In addition, suppression of Notch induced morphological changes in explant-originated cells. While NICD –over-expressed cells displayed a spindle-shaped appearance, GSI-treated cells acquired an epithelioid cobblestone-like morphology (Figure 5A). Using immunocytochemistry we showed that acquisition of epitheloid morphology was associated with reduction in N-cadherin, induction of epithelial marker E-cadherin and up-regulation of membrane-associated β-catenin (Figure 5B, Figure S6).Taken together the results of qRT-PCR, flow cytometry and immunostaining indicate that Notch stimulation promoted mesenchymal transition and differentiation of c-Kit+ and c-Kit- cells, while Notch suppression reversed mesenchymal transition. In c-Kit+ cells, Notch suppression also up-regulated the expression of pluripotency genes.

### Notch and Wnt/β-catenin Crosstalk

**Figure 6:**
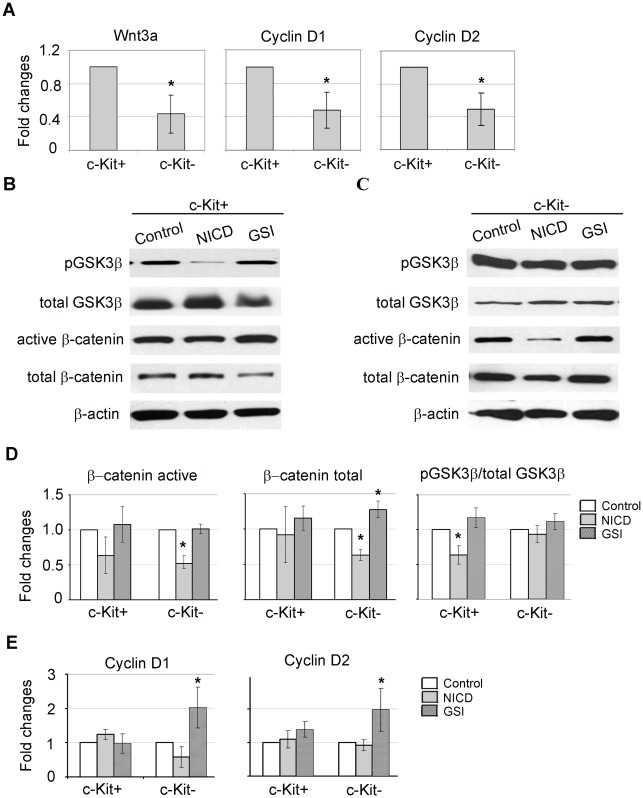
Notch modulation affects state of Wnt signaling. (**A**) Comparative qRT-PCR analysis of selected Wnt signaling-related genes in c-Kit+ and c-Kit- cells. (**B**–**E**) Notch signaling was up-regulated by over-expressing NICD-GFP adenovirus or suppressed with GSI. (**B**–**C**) Western blot of selected Wnt cascade components. β-Actin was used as a loading control. Representative images are shown. (**D**) Density of specific bands were quantified and normalized to the density of beta-actin. Level of inactive GSK3β was determined as ratio between pGSK3β (Ser 9) and total GSK3β. (**E**) qRT-PCR analysis of selected Wnt target genes in NICD-treated or GSI-treated cells. N = 5 per treatment condition. *, p<0.05.

Wnt/β-catenin cascade was recently reported to pay a role in EMT [23], [24]. Therefore, we asked whether or not Wnt/β-catenin is involved in Notch-modulated EMT via in c-Kit+ and c-Kit- cells. Compared to c-Kit-, we found that c-Kit+ cells express significantly higher levels of Wnt3a, gene coding ligand, Cyclin D1 and Cyclin D2, genes coding direct targets of canonical Wnt/β-catenin signaling (Figure 6A), suggesting that canonical Wnt/β-catenin signaling is more active in c-Kit+ compared to c-Kit- cells. Two major components of canonical Wnt cascade are β-catenin and glycogen synthase kinase 3β (GSK3β). GSK3β phosphorylates β-catenin and targets it to degradation, which ultimately maintains Wnt signaling at a low level. Inactivation of GSK3β results in accumulation and/or nuclear translocation of β-catenin. Nuclear β-catenin serves as a transcriptional factor promoting transcription of Wnt target genes [25]. In addition β-catenin regulates cell adhesion via interaction with E-cadherin. To examine more closely the state of Wnt signaling, we performed Western blot with antibodies against total GSK3β, inactive (Ser9 phosphorylated) GSK3β (pGSK3β), total β-catenin and transcriptionally active (Ser37 dephosphorylated) β-catenin. At the base level, we found no significant differences in the pGSK3β/total GSK3β ratios and in the protein levels of total and active β-catenins between c-Kit+ and c-Kit- cells (Figure S7). NICD treatment reduced pGSK3β/total GSK3β protein ratio in c-Kit+ cells, while no change were found in the levels of active and total β-catenin proteins. In c-Kit- cells, NICD treatment significantly reduced the levels of active and total β-catenin (Figure 6B–D). In contrast, Notch inhibition increased total β-catenin in c-Kit- cells (Figure 6B–D). Accumulated β -catenin in GSI-treated c-Kit- cells mostly presented in cytosolic/membrane-associated forms and possibly contributed to MET through interaction with E-cadherin (Figure 5). Furthermore, GSI treatment resulted in up-regulation of Wnt target genes Cyclin D1 and Cyclin D2 in c-Kit- cells (Figure 6E). In contrast, no significant changes in expression levels of Wnt target genes were found in GSI- or NICD- or treated c-Kit+ cells. In summary, these data showed that in c-Kit- cells Notch induction is associated with degradation of β-catenin, while Notch down-regulation stimulated β-catenin accumulation followed by up-regulation of Wnt target genes, indicating a negative feedback between Notch and Wnt/β-catenin signaling in these cells.

## Discussion

Here we showed that explants-derived cells underwent spontaneous EMT-like process in culture, during which c-Kit+ cells were generated. Furthermore, we showed that Notch activation in c-Kit+ cells further promoted EMT, while its suppression reversed EMT process leading to an activation of pluripotent genes. This EMT observation is supported by an increase in SMA+ cells, an increase in Snail gene expression, and a decrease in E-cadherin+ cells. This phenomenon cannot be explained by selective proliferation, and/or diminished apoptosis of fibroblasts, or differentiation of c-Kit+ cells to fibroblasts for the following reasons: 1) proliferation and apoptosis rates of progenitor cells and fibroblasts are similar; and 2) the number of progenitor c-Kit+ cells in the cell outgrowth remained relatively stable (Figure 1B, D, and E). The more likely explanation of the spontaneous EMT is to the release of EMT modulators such as TGF-β and/or cell-to–cell interaction between c-Kit+ cells and fibroblasts in culture. This explanation is consistent with previous reports indicating that epicardial EMT can be induced by activation of TGF-β signaling [26], [27] and epicardial cells undergo spontaneous EMT when cultured in the presence of fibroblasts [28], [29].

Furthermore, we found that approximately 50% of total outgrowth and 74% of c-Kit+ cells expressed Wt1 indicating their epicardial origin [30]. This finding is in agreement with reports showing that during development and in adult hearts after infarction, epicardial cells gave rise to cardiovascular progenitors through EMT [19], [31]–[33]. Here we found that, in native atria, epicardial cells are negative for c-Kit, while explant-derived c-Kit+ cells express epicardial marker Wt1 as well as pluripotency markers Nanog and Sox2 (Figure 2). Our results are consistent with accumulating evidences showing that mesenchymal phenotype of cells generated by EMT may represent a transient microenvironment-dependent cell state that accompanied by re-activation of fetal gene program and transformation of somatic cells to pluripotent state [34]–[37].

We found that Notch 1 receptor was expressed mainly in c-Kit+ cells, while Jagged 1 is expressed mainly in c-Kit- cells. These data suggest that in cell outgrowth, c-Kit- cells may stimulate Notch signaling in c-Kit+ progenitors via direct cell-to-cell contacts leading to mesenchymal transition and/or pluripotency gene re-activation. This observation is in agreement with reports indicating that Notch signaling stimulates EMT of epicardial-derived cells [21], [22] and maintain pluripotency in cardiac resident progenitor cells [38]. We found that suppression of Notch signaling significantly delayed cell migration out of explants and increased the percentage of c-Kit+ cells, probably due to partial inhibition of EMT in culture (Figures S3 and S4). In both c-Kit+ and c-Kit- cells activation of Notch stimulation promoted cell transition toward the mesenchymal phenotype, as indicated by increased numbers of SMA+ cells, and decreased numbers of E-cadherin+ cells. In contrast, Notch suppression reversed the mesenchymal transformation of these cells and led to epithelial-like changes in cell morphology more profoundly in c-Kit- cells (Figure 4D–F, Figure 5). Notch inhibition was associated with further up-regulation of Nanog and Sox2 pluripotency genes only in c-Kit- cells. One may hypothesize that, in c-Kit+ cells, Notch functions as a gatekeeper, providing a fine regulation of these pluripotency genes. Additional studies are needed to elucidate this hypothesis.

Our data indicates that in c-Kit- cells Wnt/β-catenin signaling is involved in EMT regulation via negative feedback with Notch signaling. This is consistent with previous studies showing that Wnt/β-catenin cascade plays a role in epicardial EMT regulation [23]. However, in c-Kit+ cells NICD over-expression led to only a slight reduction in the active β-catenin protein level and had no effect on the total β-catenin protein level and the expression level of Wnt target genes (Figure 6B–E). In addition, no effects were observed when Notch signaling was inhibited in c-Kit+ cells (Figure 6B–E). The lack of Notch modulation effect on Wnt/β-catenin cascade in c-Kit+ cells may suggest that in these cells Wnt cascade is regulated independently of Notch. Another possible explanation is that Wnt pathway lies up-stream to Notch signaling.

In conclusion, the presented study demonstrates that cardiac explant outgrowth underwent an EMT-like process in culture. Notch activation promoted this process, while Notch suppression resulted in EMT reversal. In addition, Notch suppression led to an increase in number of c-Kit+ cells and stimulated pluripotency gene expression only in c-Kit+ cells. These data are useful for optimizing the scaled-up production and phenotype modulation of progenitor cells needed for clinical applications.

## Supporting Information

Figure S1 **Proliferation (A) and apoptosis (B) in cardiac outgrowth sub-populations.** (A) ECDs were treated with BrdU following by labeling with anti-BrdU antibody conjugated with Alexa-488 and lineage specific antibodies conjugated with PE. Double positive events were detected by flow cytometry. (B) Apoptosis of EDCs sub-populations. EDCs were labeled with anti-activated caspase-3 antibody conjugated with Alexa-488 and lineage specific antibodies conjugated with PE. Double positive events were detected by flow cytometry. Representative histograms are shown.
(TIF)

Figure S2 **Purity of c-Kit+ (A) and c-Kit- (B) cell subsets were confirmed by immunocytochemistry.** Cells were labeled with anti c-Kit antibody followed by secondary antibody conjugated with Alexa 488 (green). Scale bar, 20 µm.
(TIF)

Figure S3 **Cardiac explants cultured in presence of GSI exhibited significant delay in generation of cell outgrowth.** N = 20.
(TIF)

Figure S4 **GSI addition to explant in explant culture media induced expression of c-Kit and Wt1 markers in EDCs.** EDCs derived from control and GSI-treated (GSI) explants were collected 21 days after culturing and subjected to flow cytometry.
(TIF)

Figure S5 **C-Kit+ and c-Kit- cells were treated to up-regulate (NICD) or suppress (GSI) Notch signaling and analyzed by flow cytometry.** FACS analysis of control (black), NICD- (blue) and GSI-treated cells (green). Representative histograms are shown. For a negative control, isotype IgG was used instead of primary antibody (grey).
(TIF)

Figure S6 **Suppression of Notch reversed mesenchymal phenotype of C-Kit+ cells.** GSI was added to cell culture media to suppress Notch signaling. Representative images of c-Kit+ cells are shown. (A) Transmitted light images demonstrate changes in cell morphology upon treatment with NICD or GSI. (B) Cells were labeled with antibodies to E-cadherin (green), N-cadherin (red) or ?-catenin (green) as indicated. Nuclei were counterstained with DAPI (blue). Scale bars, 100 µm (A) or 20 µm (B).
(TIF)

Figure S7 **Western blot analysis of Wnt signaling components in c-Kit+ and c-Kit- cells.** Density of specific bands were quantified and normalized to the density of beta-actin. Level of inactive GSK3β was determined as ratio between pGSK3β (Ser 9) and total GSK3β. N = 5.
(TIF)

Table S1 **Primary antibodies.** ICC, immunocytochemistry; IHC, immunohistochemistry; FC, flow cytometry; WB, Western blotting.
(DOCX)

Table S2 **Gene expression analysis of Notch modulated c-Kit+ and c-Kit- cells.** ↓ Gene expression significantly decreased compared to control; ↑ Gene expression significantly increased compared to control; ↔ No significant differences.
(DOCX)

Table S3 **FACS analysis of Notch-modulated c-Kit+ and c-Kit- cells.** ↓ Gene expression significantly decreased compared to control; ↑ Gene expression significantly increased compared to control; ↔ No significant differences.
(DOCX)
