# Supplementary figures and images for: Cardiac Explant-Derived Cells Are Regulated by Notch-Modulated Mesenchymal Transition
Source: PLoS One. 2012 May 25;7(5):e37800. doi: 10.1371/journal.pone.0037800 (PMC3360598; doi:10.1371/journal.pone.0037800)

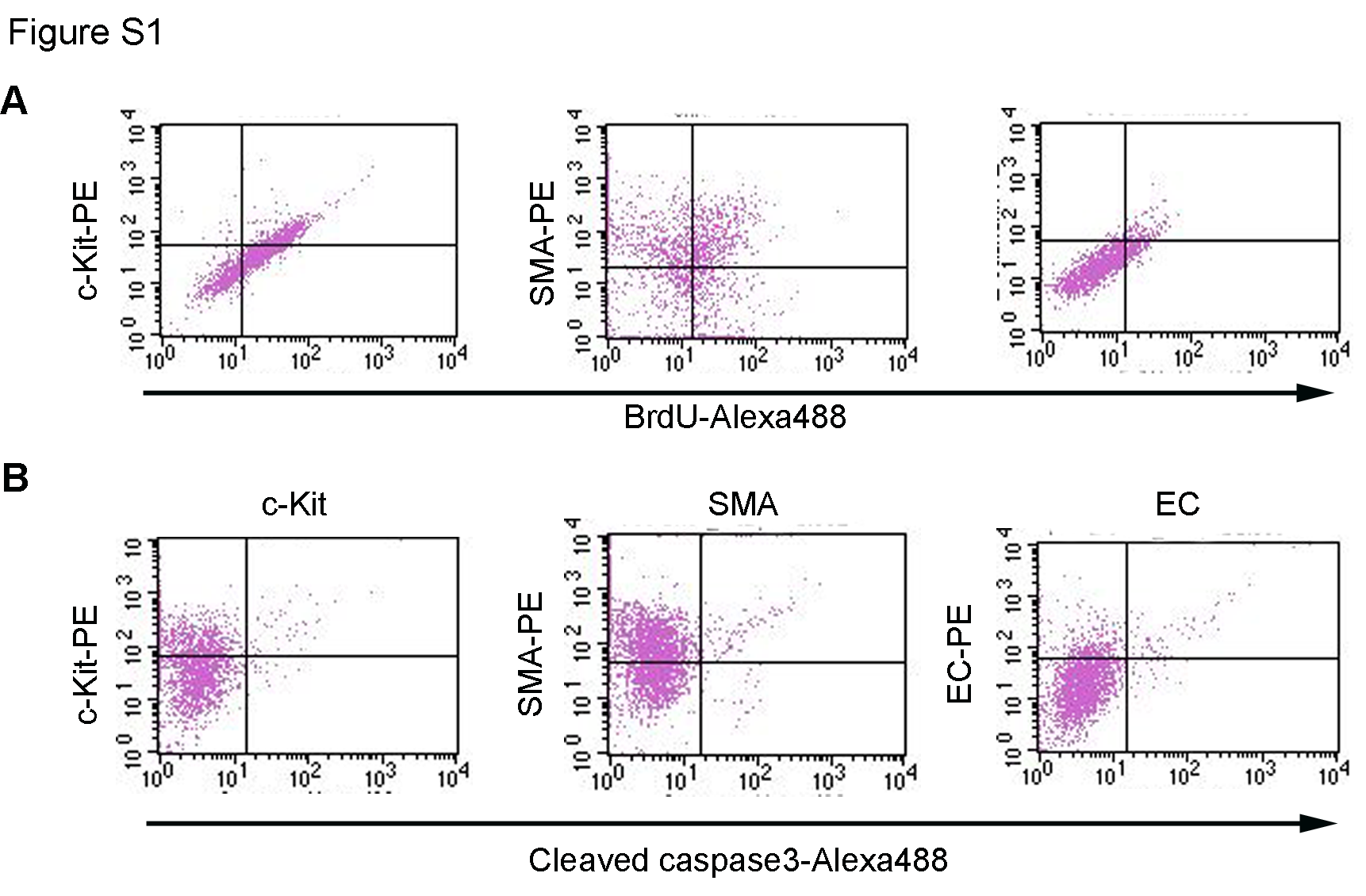

Supplement: Figure S1 — Proliferation (A) and apoptosis (B) in cardiac outgrowth sub-populations. (A) ECDs were treated with BrdU following by labeling with anti-BrdU antibody conjugated with Alexa-488 and lineage specific antibodies conjugated with PE. Double positive events were detected by flow cytometry. (B) Apoptosis of EDCs sub-populations. EDCs were labeled with anti-activated caspase-3 antibody conjugated with Alexa-488 and lineage specific antibodies conjugated with PE. Double positive events were detected by flow cytometry. Representative histograms are shown. (TIF) [file pone.0037800.s001.tif]

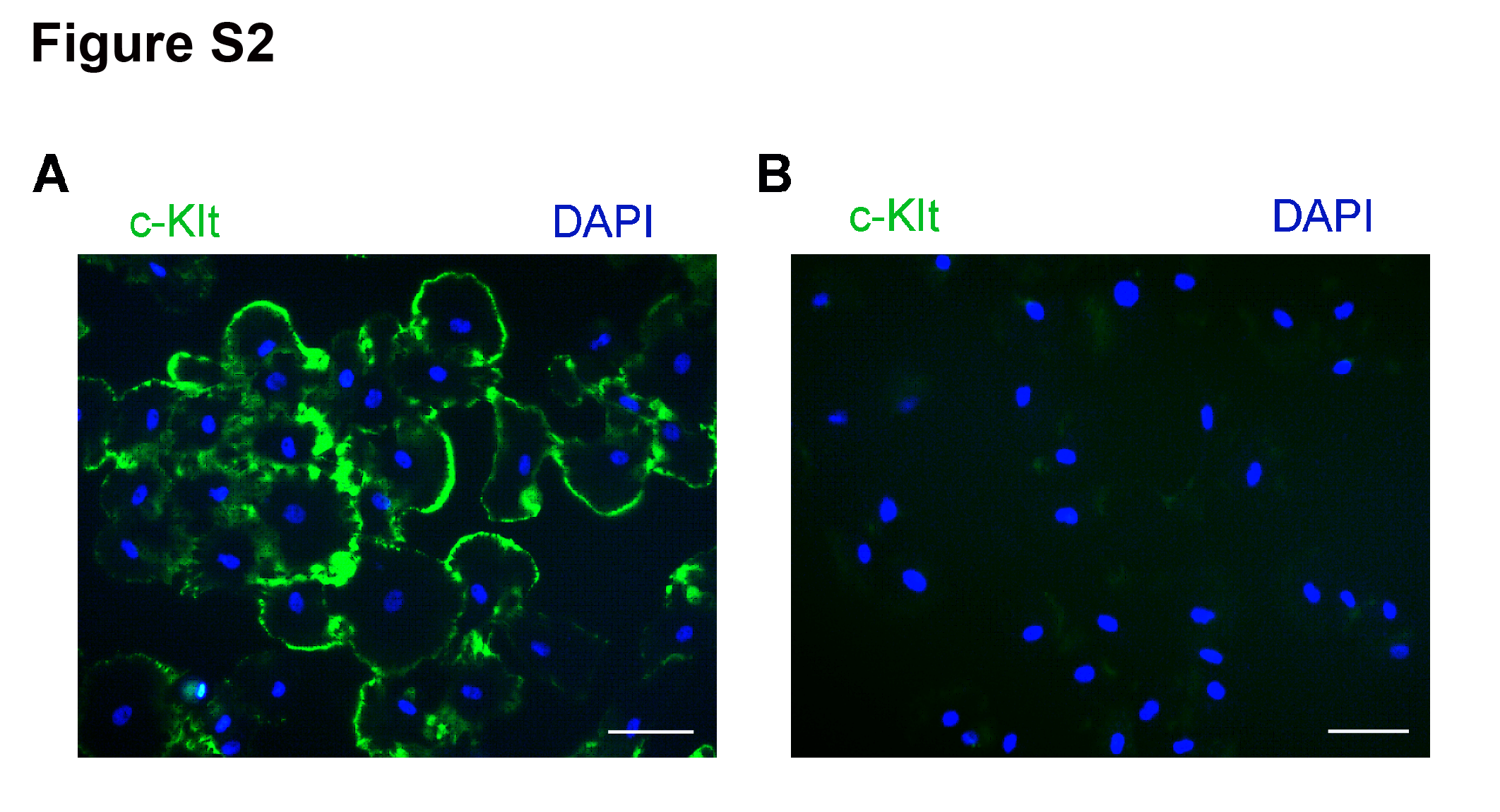

Supplement: Figure S2 — Purity of c-Kit+ (A) and c-Kit- (B) cell subsets were confirmed by immunocytochemistry. Cells were labeled with anti c-Kit antibody followed by secondary antibody conjugated with Alexa 488 (green). Scale bar, 20 µm. (TIF) [file pone.0037800.s002.tif]

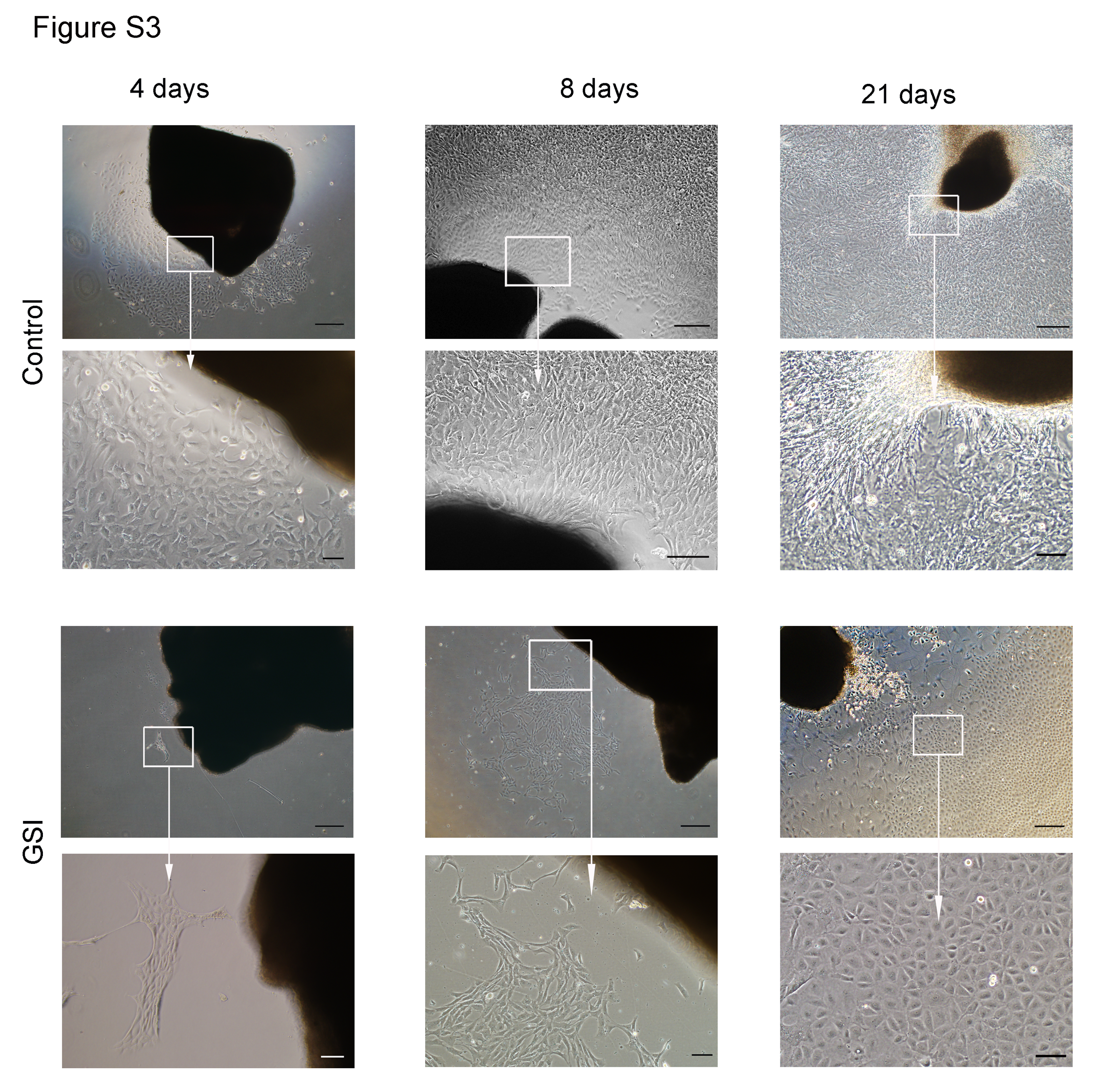

Supplement: Figure S3 — Cardiac explants cultured in presence of GSI exhibited significant delay in generation of cell outgrowth. N = 20. (TIF) [file pone.0037800.s003.tif]

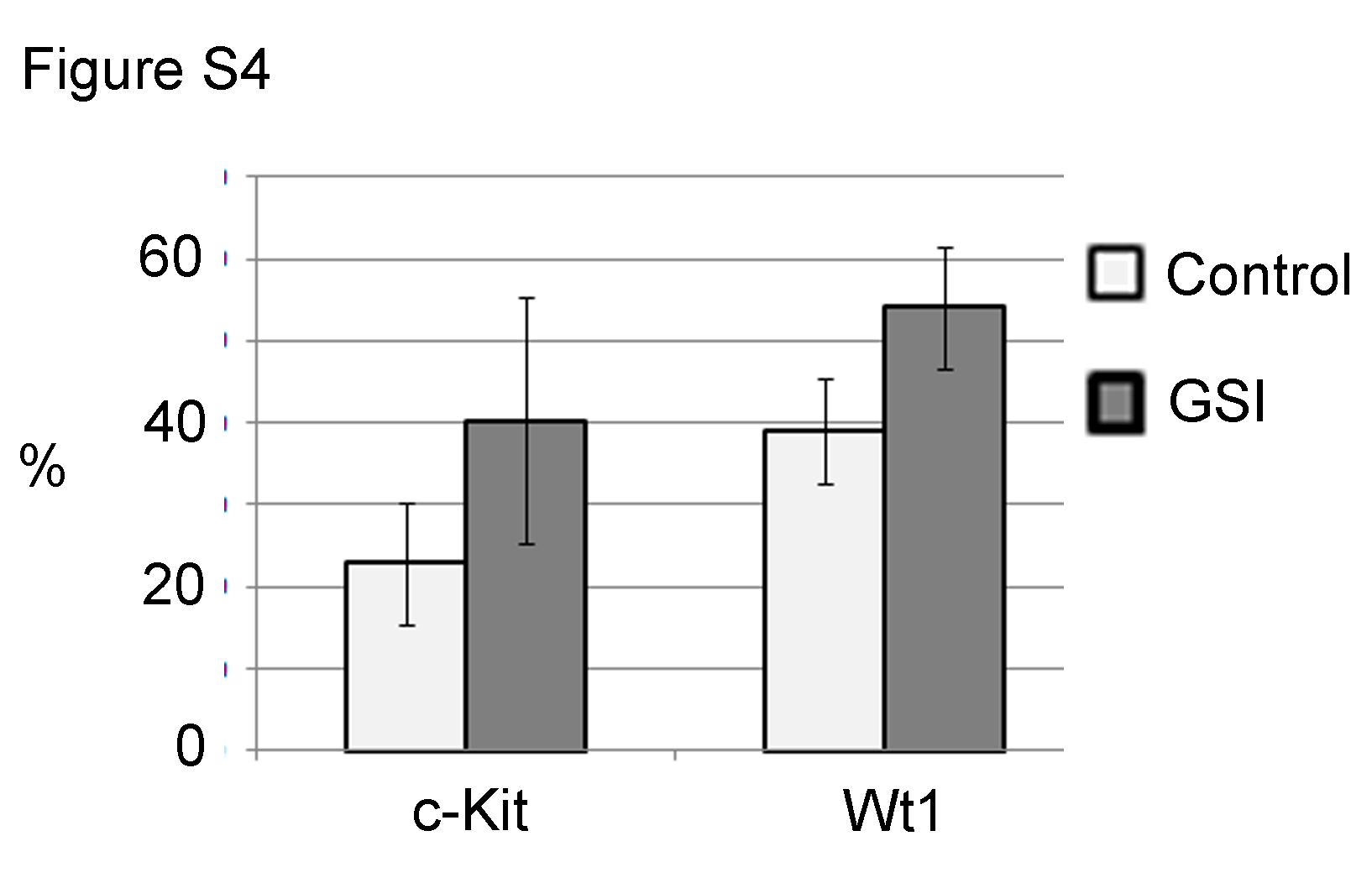

Supplement: Figure S4 — GSI addition to explant in explant culture media induced expression of c-Kit and Wt1 markers in EDCs. EDCs derived from control and GSI-treated (GSI) explants were collected 21 days after culturing and subjected to flow cytometry. (TIF) [file pone.0037800.s004.tif]

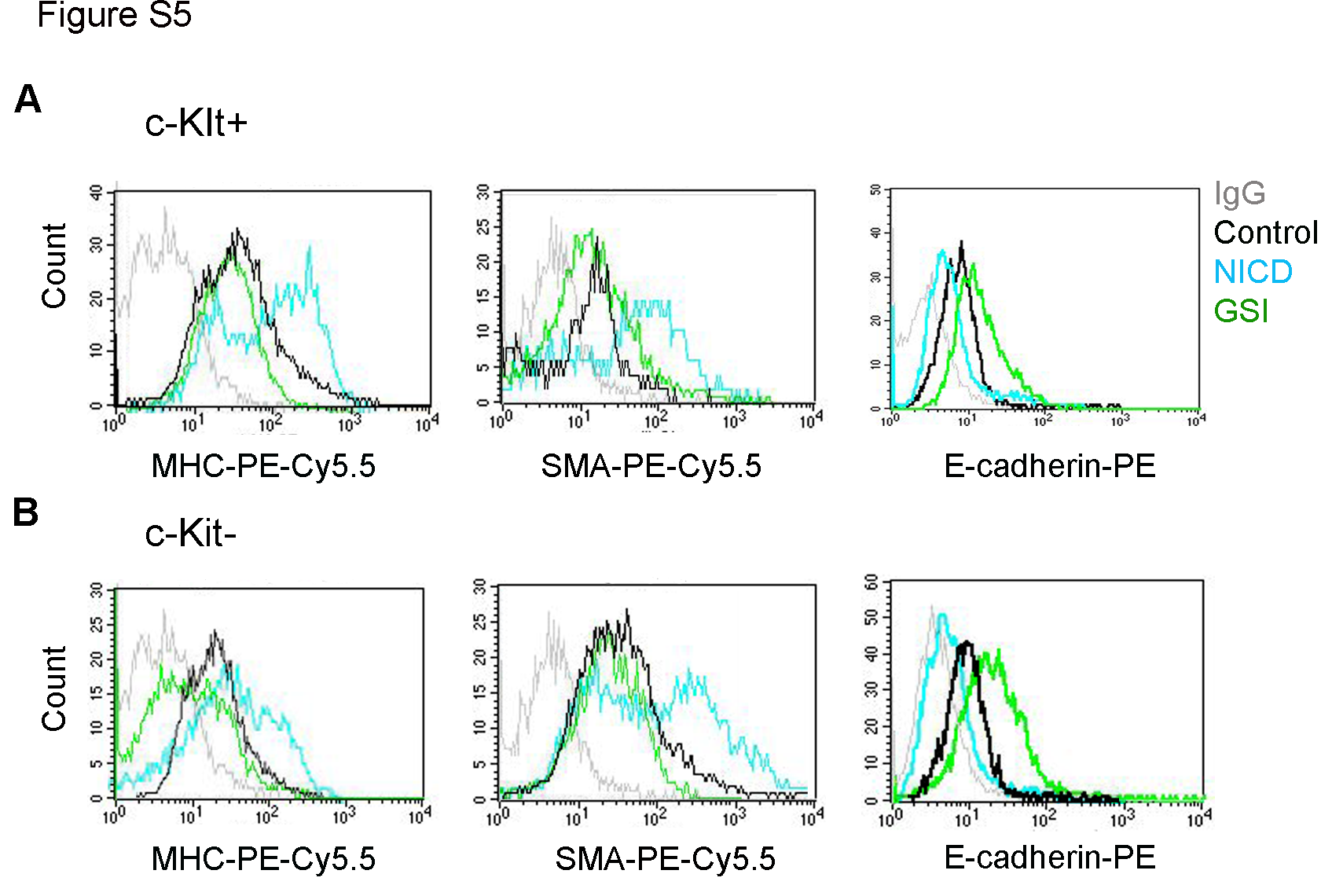

Supplement: Figure S5 — C-Kit+ and c-Kit- cells were treated to up-regulate (NICD) or suppress (GSI) Notch signaling and analyzed by flow cytometry. FACS analysis of control (black), NICD- (blue) and GSI-treated cells (green). Representative histograms are shown. For a negative control, isotype IgG was used instead of primary antibody (grey). (TIF) [file pone.0037800.s005.tif]

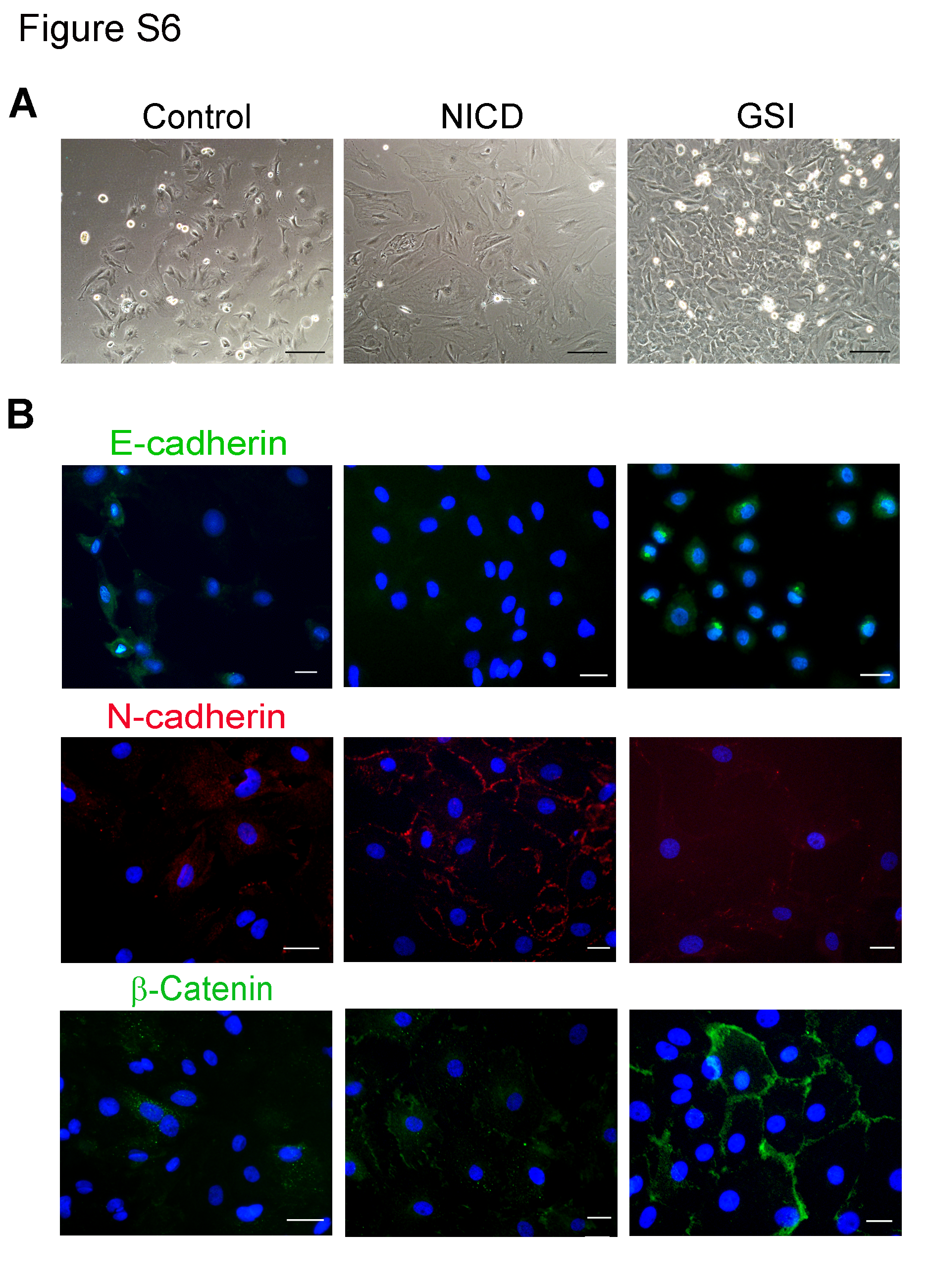

Supplement: Figure S6 — Suppression of Notch reversed mesenchymal phenotype of C-Kit+ cells. GSI was added to cell culture media to suppress Notch signaling. Representative images of c-Kit+ cells are shown. (A) Transmitted light images demonstrate changes in cell morphology upon treatment with NICD or GSI. (B) Cells were labeled with antibodies to E-cadherin (green), N-cadherin (red) or ?-catenin (green) as indicated. Nuclei were counterstained with DAPI (blue). Scale bars, 100 µm (A) or 20 µm (B). (TIF) [file pone.0037800.s006.tif]

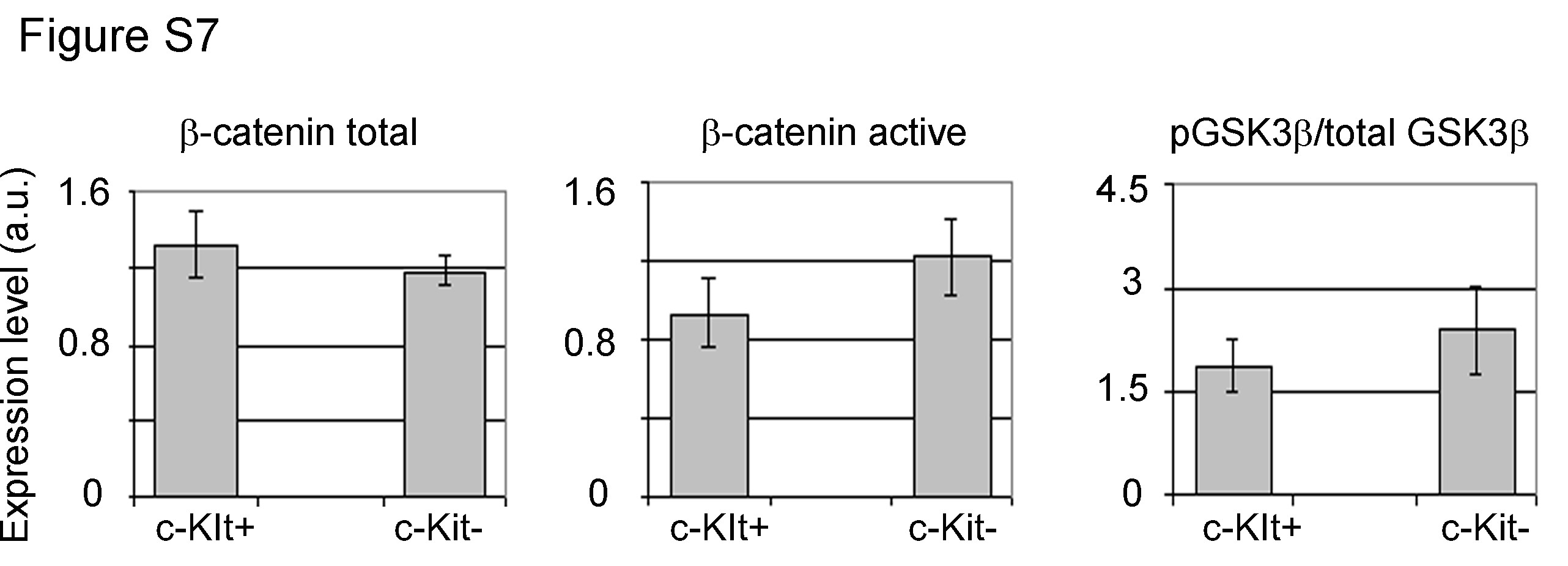

Supplement: Figure S7 — Western blot analysis of Wnt signaling components in c-Kit+ and c-Kit- cells. Density of specific bands were quantified and normalized to the density of beta-actin. Level of inactive GSK3β was determined as ratio between pGSK3β (Ser 9) and total GSK3β. N = 5. (TIF) [file pone.0037800.s007.tif]
