# Supplementary material for: Cardiac Explant-Derived Cells Are Regulated by Notch-Modulated Mesenchymal Transition
Source: PLoS One. 2012 May 25;7(5):e37800. doi: 10.1371/journal.pone.0037800 (PMC3360598; doi:10.1371/journal.pone.0037800)
Supplement: Table S1 — Primary antibodies. ICC, immunocytochemistry; IHC, immunohistochemistry; FC, flow cytometry; WB, Western blotting. (DOCX) [file pone.0037800.s008.docx]

**Table S1 Primary antibodies**

| **Antibody** | **Host** | **Detection** | **Source** |
| --- | --- | --- | --- |
| c-Kit | rabbit | ICC, FC | Santa-Cruz Biotech |
| α-Myosin heavy chain | mouse | ICC, FC | Abcam |
| α-Smooth muscle actin | mouse | ICC, FC | Abcam |
| Wt1 | rabbit | FC | Abcam |
| FSP-1 | rabbit | ICC, FC | Abcam |
| E-cadherin | rabbit | ICC, IHC, FC | Santa-Cruz Biotech |
| N-cadherin | mouse | ICC, IHC | Santa-Cruz Biotech |
| Activated (cleaved) caspase-3 | rabbit | FC | Cell Signaling Technologies |
| Notch1 | goat | ICC | Santa-Cruz Biotech |
| Jagged1 | goat | ICC | Santa-Cruz Biotech |
| Notch activated domain (NICD) | rabbit | ICC, WB | Cell Signaling Technologies |
| GSK3β | rabbit | WB | Cell Signaling Technologies |
| phGSK3β | rabbit | WB | Cell Signaling Technologies |
| Active β-catenin | mouse | WB | Millipore |
| Total β-catenin | mouse | WB | Santa-Cruz Biotech |
| β-Actin | rabbit | WB | Cell Signaling Technologies |

**Abbreviation:** ICC, immunocytochemistry; IHC, immunohistochemistry; FC, flow cytometry; WB, Western blotting.
