# Supplementary material for: Cardiac Explant-Derived Cells Are Regulated by Notch-Modulated Mesenchymal Transition
Source: PLoS One. 2012 May 25;7(5):e37800. doi: 10.1371/journal.pone.0037800 (PMC3360598; doi:10.1371/journal.pone.0037800)
Supplement: Table S2 — Gene expression analysis of Notch modulated c-Kit+ and c-Kit- cells. ↓ Gene expression significantly decreased compared to control; ↑ Gene expression significantly increased compared to control; ↔ No significant differences. (DOCX) [file pone.0037800.s009.docx]

**Table S2. Gene expression analysis of Notch modulated c-Kit+ and c-Kit- cells**

**C-Kit+**

| **Gene** | **Response to NICD*** | **Response to GSI*** |
| --- | --- | --- |
| Slug | ↑ | ↔ |
| MHC | ↑ | ↔ |
| Sox2 | ↔ | ↑ |
| Nanog | ↓ | ↑ |
| Wt1 | ↔ | ↑ |

**C-Kit- cells**

| **Gene** | **Response to NICD** | **Response to GSI** |
| --- | --- | --- |
| Slug | ↔ | ↔ |
| MHC | ↑ | ↔ |
| Sox2 | ↔ | ↔ |
| Nanog | ↔ | ↔ |
| Wt1 | ↔ | ↔ |

↓ Gene expression significantly decreased compared to control;

↑ Gene expression significantly increased compared to control;

↔ No significant differences.
